# The relationship between Fibroblast Growth Factor-21 and characteristic parameters related to energy balance in dairy cows

**DOI:** 10.1186/s12917-015-0585-4

**Published:** 2015-10-24

**Authors:** Chuang Xu, Qiushi Xu, Yuanyuan Chen, Wei Yang, Cheng Xia, Hongjiang Yu, Kuilin Zhu, Taiyu Shen, Ziyang Zhang

**Affiliations:** College of Animal Science and Veterinary Medicine, Heilongjiang Bayi Agricultural University, Daqing, 163319 People’s Republic of China; Synergetic Innovation Center of Food Safety and Nutrition, Northeast Agricultural University, Harbin, 150030 People’s Republic of China

**Keywords:** Serum FGF-21, Negative energy balance (NEB), Blood metabolites

## Abstract

**Background:**

Negative energy balance (NEB) is a common pathological foundation of ketosis and fatty liver. Liver and fat tissue are the major organs of lipid metabolism and take part in modulating lipid oxidative capacity and energy demands, which is also a key metabolic pathway that regulates NEB develop during perinatal period. Fibroblast growth factor-21 (FGF-21) is a recently discovered protein hormone that plays an important and specific regulating role in adipose lipid metabolism and liver gluconeogenesis for human and mouse. Our aim is to investigate the variation and relationship between serum FGF-21 concentration and characteristic parameters related to negative energy balance in different energy metabolism state.

**Methods:**

In this research, five non-pregnant, non-lactating Holstein–Friesian dairy cows were randomly allocated into two groups. The interventions were a controlled-energy diet (30 % of maintenance energy requirements) and a moderate-energy diet (120 % of predicted energy requirements) that lasted for the duration of the experiment. We measured biochemical parameters, serum FGF-21, leptin and insulin levels by commercial ELISA kits.

**Results:**

The results showed that serum FGF-21 levels were significantly higher in both groups treated with a controlled-energy diet, while FGF-21 levels in both groups treated with moderate-energy diet were low. FGF-21 levels exhibited a significant positive correlation with serum leptin levels, while an inverse relationship was found between FGF-21 and blood glucose and β-hydroxybutyrate acid (BHBA) levels.

**Conclusion:**

An increase in FGF-21 levels after a controlled-energy diet treatment may contribute to a positive metabolic effect which could result in a new theoretical and practical basis for the preventive strategy of dairy cows with NEB.

## Background

Negative energy balance (NEB) which is characterized by gluconeogenesis and lipodystrophy is the pivotal point and common pathological foundation of ketosis and fatty liver. Liver and fat tissue are the major organs of lipid metabolism and take part in modulating lipid oxidative capacity and energy demands, which is also a key metabolic pathway that regulates NEB development during the perinatal period.

FGF-21 is a recently discovered protein hormone that is found commonly in liver. FGF-21 plays an important role in regulating adipose lipid metabolism and liver gluconeogenesis in humans and mice. Recent experimental studies on mice have identified brown adipose tissue as one of the key sources of FGF-21 when exposed to cold conditions. It is mainly through Beta adrenaline and cyclic adenosine monophosphate, which mediates norepinephrine, and, finally an activated protein kinase to mediate the process of FGF-21 gene transcription and FGF-21 protein release [1, 2]. Experimental studies have also identified that FGF-21 had a protective effect on Diet Induced Obesity (DIO) mice, and an increase in energy expenditure and fat utilization were found in further studies [3–5]. The expression of FGF-21 in the murine liver is tightly regulated nutritionally. It is increased by starvation and ketogenic state and decreased by refeeding [6].

Human studies have shown that serum FGF-21 levels were significantly higher in type 2 diabetes mellitus (T2DM) patients relative to healthy controls, and this suggests the possibility of direct positive metabolic effects of FGF-21 in humans [7]. Similarly, a positive relationship of serum FGF21 levels with liver lipid content and Body Mass Index (BMI) [7] and an inverse relationship of FGF-21 with serum adiponectin levels were found in non-alcoholic fatty liver disease (NAFLD) patients. It may indicate that FGF-21 regulates mammals in different energy states [8–10].

It was clear that the study of the new regulatory factor FGF-21 on human and mice yielded important results. However, the state of FGF-21 research in dairy cows is just beginning. In contrast to these studies of humans and other mammals, very little is known about the regulation of FGF-21 in dairy cows. Recent experimental studies have found that the content of FGF-21 increased rapidly postpartum and after that maintained a lower level during the perinatal period [11, 12]. However, to our knowledge, no information about the dynamic changes in FGF-21 levels after dietary intervention in dairy cows is available. In view of the above mentioned research status of FGF-21, there are some key scientific problems that need clarification in the relationship between FGF-21 and energy metabolism in dairy cow:

1. No information about how FGF-21 exerts a direct influence on energy intake is available, although variation in FGF-21 levels during the perinatal period has been reported in dairy cows.
2. There are still some theoretical issues to be resolved on the effect that changes in energy intake have on FGF-21 levels, and the effect that energy intake has on FGF-21 levels.

To this end, our aim is to confirm a hypothesis that FGF-21 might play the above-mentioned role in dairy cows with NEB. Non-pregnant, non-lactating dairy cows will be allocated randomly to our experiments to recreate the energy deficit of perinatal period. By means of biochemical technologies and clinical investigation, our preliminary study will document the changes in FGF-21 concentration in the blood during energy insufficiency and when being overfed, investigate the relationship between FGF-21 and some characteristic parameters related to energy metabolism in the blood, and reveal a regulation for FGF-21 on energy balance in dairy cows. Our work may provide a new theoretical and practical basis for preventing NEB in dairy cows.

## Methods

### Ethics statement

The study was approved by the farm owner and all experimental animals were conducted according to the International Guiding Principles for Biomedical Research. The protocol was approved by the Committee on the Ethics of Animal Experiments of the Heilongjiang Bayi Agricultural University.

### Herd and cow selection

In northeastern China (MiShan, Heilongjiang, China), commercial dairy herds from a large dairy farm were selected to participate in the study. Five non-pregnant, non-lactating Holstein–Friesian dairy cows (18 ± 4 months of age) were allocated randomly based on specific condition needs of compliance with the study protocol. All dairy cows were in good health.

### Animal management

**Fig. 1:**
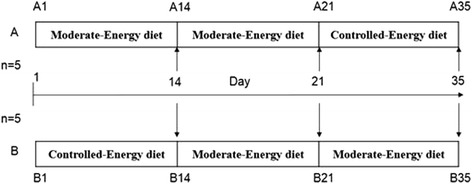
Design of trial

This study included ten Holstein cows, five dairy cows were assigned randomly to two groups for the experiment. Group A was treated with a moderate-energy diet (120 % of predicted energy requirements) in the primary stage of the experiment and then they were treated with a controlled-energy diet (30 % of maintenance energy requirements) afterwards. Group B was treated with a controlled-energy diet (30 % of maintenance energy requirements) first and then they were treated with a moderate-energy diet (120 % of predicted energy requirements) in the later stage. Each level of nutrition was achieved by feeding appropriate amounts of a single TMR (Total Mixed Rations). The TMR composition was 1.48 Mcal (Mega-calorie) net energy of lactation (NE_L_) and 160 g of crude protein per kilogram of dry matter (DM). Feeding duration for both the controlled-energy and moderate-energy diets was 14 days; both groups were given a moderate-energy diet (120 % of predicted energy requirements) for 7 days before the change in diet (Fig. 1).

### Sample collection

Blood samples were collected from tail vein in early morning before receving diet every day. After blood collection, tubes without anticoagulant were placed in an icebox and carried to the laboratory within 1 h of collection, after which they were placed at room temperature for 30 min, centrifuged at 3000 g for 15 min, and then stored at −20 °C until analysis.

### Blood metabolites and hormones

Concentrations of FGF-21 were measured using a RD Human Fibroblast Growth Factor-21 ELISA kit (R&D Systems Inc. Minneapolis, MN, USA) on an automatic biochemistry analyzer (JianCheng Biological Engineering Research Institute, Nanjing, China). Glucagon (GC) was measured with a RD Bovine Glucagon ELISA kit, growth hormone (GH) was measured with a RD Bovine Growth hormone ELISA kit, and insulin (INS) was measured with a RD Bovine INS ELISA kit. All biochemical parameters were analysed on a Huadong electronic DG5033A microplate reader (Huadong, Nanjing, China). Blood glucose (GLU), serum levels of triglycerides (TG) and nonesterified fatty acid (NEFA) were measured directly by using an automatic biochemistry analyzer.

### Data collation and statistical analyses

Data in this study were collated and initially analyzed using Excel 2013 (Microsoft Corp., Redmond, WA, USA). Descriptive and graphical analyses were carried out to verify the data. When appropriate, we used IBM SPSS19.0 software (SPSS Inc. Chicago, IL) to analyze the data. The results are expressed as means ± standard error means (SEM). Changes in FGF-21 levels and hormonal parameters between two groups were evaluated by an Independent Sample *T*-test. One-way ANOVA was used to compare data from different time periods between the two groups, and multiple testing was corrected using the LSD (Least Significant Difference) method (Equal Variances Assumed) or the Dunnett T3 (Equal Variance not assumed). Pearson correlation tests were used to calculate the relationships between FGF-21 levels and other parameters. Pearson’s correlation analysis and linear regression analysis were used to analyze the correlation of BHBA and serum FGF-21. In data testing, a P value <0.05 was considered statistically significant.

## Results

The body weight changes in dairy cow are shown in Table 1. In this study, the body weight of dairy cows in both group gained significantly when treated with a moderate-energy diet and the loss of body weight were noticed when dairy cows were treated with a controlled-energy diet.

**Table 1:** Variation in dairy cow weight changes between Group A and Group B

Group	Time point (Days)			
	1	14	21	35
A	337.80 ± 25.49	358.40 ± 24.39*	368.20 ± 22.74*	356.00 ± 22.33*
B	332.00 ± 29.43	318.60 ± 29.08*	328.60 ± 28.28	347.40 ± 25.10*

Unit of measurement: KG. Group A, treated with a moderate-energy diet in primary stage and treated with a controlled-energy diet in later stage. Group B, treated with a controlled-energy diet in primary stage and treated with a moderate-energy diet in later stage. Values are means ± SEM. Statistical significance is from one-way anova. *P < 0.05 vs. Baseline dairy cow weight data in same group

### The relationship between serum FGF-21 and energy intake level

**Fig. 2:**
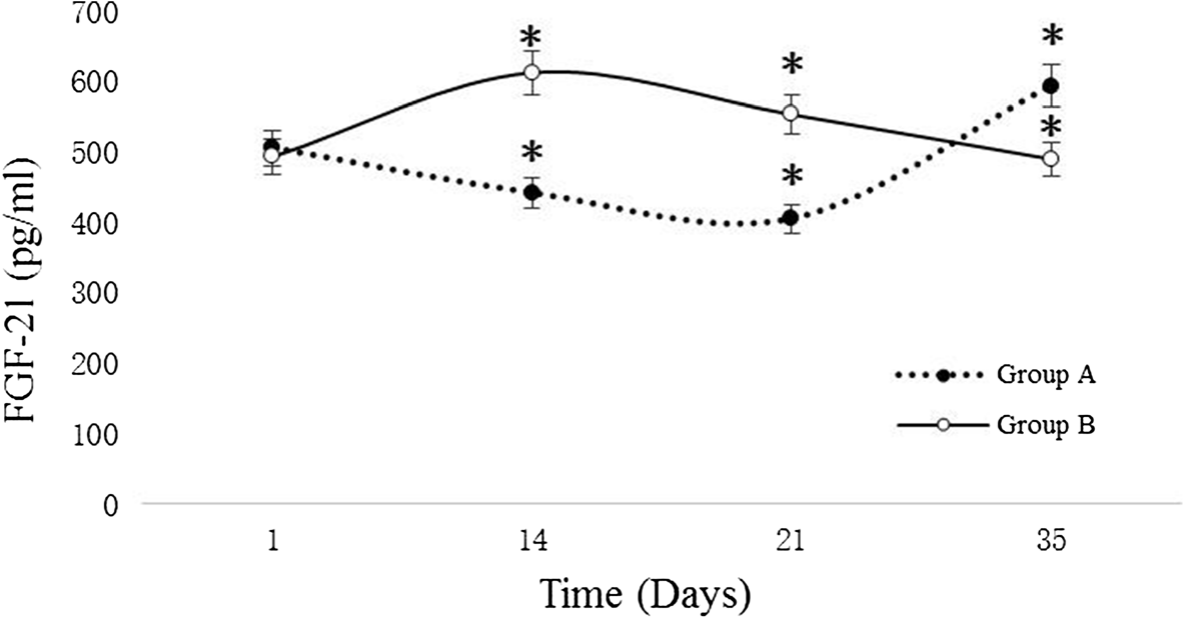
Variation of Serum FGF-21 changes between Group A and Group B. Changes in FGF21 concentrations during the variation of energy intake of control Group A (dash line with filled circles), and changes in FGF21 concentrations during the variation of energy intake for control Group B (solid line with open circles). Statistical significance is based on a one-way anova: *P < 0.05 vs. baseline FGF-21 value in same group

Serum FGF-21 levels at different time point of two groups are shown in Table 2. As expected, serum FGF-21 levels changed significantly after the variation in energy intake. The variation tendency of FGF-21 is shown in Fig. 2. It showed that Serum FGF-21 levels were closely related to energy intake level.

**Table 2:** Variation in Serum FGF-21 changes between Group A and Group B

Group	Time point (Days)			
	1	14	21	35
A	506.48 ± 48.01	442.12 ± 67.65*	405.39 ± 47.56*	594.24 ± 40.31*
B	493.72 ± 90.62	612.89 ± 80.70*	553.93 ± 121.38*	490.54 ± 62.19*

Group A, treated with a moderate-energy diet in primary stage and treated with a controlled-energy diet in later stage. Group B, treated with a controlled-energy diet in primary stage and treated with a moderate-energy diet in later stage. Values are means ± SEM. Statistical significance is from one-way anova. *P < 0.05 vs. Baseline FGF-21 data in same group

### Effect of energy intake on serum levels of FGF-21

We gathered clinical trial data for the two groups on eight days over a 14-day period for dairy cows in two groups that were fed two different energy-state diets. Serum FGF-21 levels decreased significantly in Group A, however, in dairy cows fed a moderate-energy diet in Group B, serum FGF-21 levels increased significantly (Table 3). There were no significant differences (*P* > 0.05) in serum FGF-21 levels in the initial experiment phase (1–4 days) between the two groups. As the experiment went on, serum FGF-21 levels between two groups assumed significant differences (*P* <0.05). Gradually lower serum FGF-21 levels were found in Group A, which was fed a moderate-energy diet. However, serum FGF-21 levels in Group B gradually peaked. We found that serum FGF-21 levels significantly declined in the high diet energy-state and increasing FGF-21 levels were found in the low energy-state diet.

**Table 3:** The effect of energy intake on Serum FGF-21 concentration over a 14-day period

Experiment days	Group	
	A	B
1	506.48 ± 48.01	493.72 ± 90.62
2	499.62 ± 58.79	475.33 ± 21.39
4	497.43 ± 86.13*	508.45 ± 94.74*
6	483.81 ± 54.40*	524.01 ± 49.61*
8	478.35 ± 111.16*	526.83 ± 94.43*
10	456.91 ± 44.47**	556.11 ± 83.56*
12	447.80 ± 54.94*	595.51 ± 74.81*
14	442.12 ± 67.65**	612.89 ± 80.70*

Group A, treated with a moderate-energy diet; Group B, treated with a controlled-energy diet. Values are means ± SEM. Statistical significance is from an Independent Sample *T* test. *P < 0.05 vs. Baseline FGF-21 data in same group

### The relationship between metabolic characteristics and FGF-21 levels under NEB

Experimental animals fed a controlled-energy diet were in the initiation phase of NEB. This is to say that experimental animals in Group A were experiencing NEB at day 21–35 and Group B animals were in NEB at day 1–14 after the experiment commenced. The biochemical and hormonal characteristics of two groups at four timings are shown in Table 4 and Table 5. The relationship between serum FGF-21 levels and metabolic characteristics in NEB in different experimental phases of the two groups are shown in Table 6. Serum FGF-21 levels were showed a significant positive correlation with serum leptin levels, while we obtained a negative correlation of FGF-21 with blood glucose and BHBA levels. On the contrary, FGF-21 levels were not significantly related to serum glucagon, triglyceride, non-esterified fatty acid, or insulin levels.

**Table 4:** Variation in Serum characteristic parameters changes in Group A

Parameters	Time point (Days)			
	A1	A14	A21	A35
Glc (mmol/L)	4.10 ± 0.43	4.34 ± 0.31*	4.96 ± 0.52*	3.89 ± 0.31*
BHBA (μmol/L)	640.73 ± 68.52	698. 63 ± 82.14*	760.63 ± 76.40*	615.73 ± 88.76
TG (mmol/L)	0.07 ± 0.03	0.09 ± 0.05	0.11 ± 0.05	0.08 ± 0.03
NEFA (μmol/L)	318.27 ± 32.65	321.52 ± 43.31	352.32 ± 22.58	298.27 ± 58.31
GC (pg/mL)	545.87 ± 130.54	550.47 ± 96.20	553.87 ± 83.24	563.87 ± 143.84
INS (mIU/L)	10.13 ± 3.86	11.38 ± 3.97	15.36 ± 5.23*	10.09 ± 3.15
LP (μg/L)	304.38 ± 29.54	282.59 ± 39.96*	268.34 ± 28.22*	312.89 ± 39.96
GH(ng/mL)	5.68 ± 1.86	5.47 ± 1.44	5.40 ± 1.33	5.63 ± 1.20

Values are means ± SEM. Statistical significance is from one-way anova. *P < 0.05 vs. Baseline Serum characteristic parameters data in same group

**Table 5:** Variation in Serum characteristic parameters changes in Group A

Parameters	Time point (Days)			
	B1	B14	B21	B35
Glc (mmol/L)	4.16 ± 0.82	3.85 ± 0.34*	4.11 ± 0.51	4.87 ± 1.22*
BHBA (μmol/L)	620.52 ± 76.51	601.39 ± 55.61*	631.75 ± 89.37*	813.46 ± 81.78*
TG (mmol/L)	0.06 ± 0.03	0.08 ± 0.02	0.10 ± 0.04	0.10 ± 0.02
NEFA (μmol/L)	351.28 ± 64.23	334.85 ± 43.54	345.44 ± 28.22*	368.56 ± 78.52
GC (pg/mL)	553.87 ± 134.66	586.93 ± 93.55	516.23 ± 83.22	563.62 ± 111.21
INS (mIU/L)	11.25 ± 3.44	11.24 ± 3.59	13.07 ± 3.38*	16.37 ± 4.16*
LP (μg/L)	310.83 ± 38.58	313.49 ± 66.13	289.64 ± 75.43*	274.46 ± 42.58*
GH(ng/mL)	5.51 ± 1.64	5.68 ± 1.42	5.47 ± 1.44	5.54 ± 1.37

Values are means ± SEM. Statistical significance is from one-way anova. *P < 0.05 vs. Baseline Serum characteristic parameters data in same group

**Table 6:** The relationships of serum FGF-21 levels with biochemical and hormonal parameters during different experimental phases

Factor	Experimental phase				
	A1-A21	A21-A35	B1-B14	B14-B35	A + B
Glc	−0.402*	−0.353*	−0.314*	−0.376*	−0.385*
BHBA	−0.432*	−0.362*	−0.437*	−0.333*	−0.357*
GC	0.134	0.260	0.160	0.211	0.154
TG	0.017	0.115	0.211	0.185	0.145
NEFA	−0.122	−0.088	−0.168	−0.183	−0.133
INS	−0.235	−0.134	−0.162	−0.248	−0.286
LP	0.682*	0.674*	0.621*	0.564*	0.628*

The correlations were calculated in different experimental phases and a combined experimental sample of two experimental groups. Results are based on a Pearson correlation test; * = significant difference between the FGF-21 and the specific indicator in the same row (*P* <0.05). No mark in the same row means there was not a significant difference between treatments (*P* >0.05)

## Discussion

Our experiment makes references to perinatal period model reported by Schoenberg [11], and our research group adequately improve the design of experiment to study the relationship between serum FGF-21 concentration and characteristic parameters related to energy balance in different energy metabolism states. Our study demonstrated that dairy cows that were under a controlled-energy diet significantly increased their serum FGF-21 levels, but for dairy cows that were under a moderate-energy diet, serum FGF-21 levels decreased significantly. FGF-21 levels of dairy cows feeding controlled-energy diet was significantly higher than dairy cows feeding moderate-energy diet. At the onset of the experiments (1–4 days), serum FGF-21 levels showed no significant change. Similarly, other experiments found that short-term fasting (12–48 h) had no effect on serum FGF-21 levels. However, an increase in acetobutyric acid level induced a significant increase in ketone bodies, and resulted in a 74 % increase in serum FGF-21 levels after 7 days of fasting [13]. Our study showed a significant decrease in serum FGF-21 levels in dairy cows under a moderate-energy diet. Collectively, this finding is in agreement with previous studies that serum FGF-21 levels and FGF-21 gene expression in liver significantly decreased when cows were over-fed [14, 15]. In animal models, obesity and T2DM have decreased serum FGF-21 levels and reduced FGF-21 gene expression [16] and, therefore, proposed that obesity is an FGF21-resistant state [10]. Dairy cows under a controlled-energy diet showed a significant increase in serum FGF-21 levels, which can be interpreted as a response to a low-energy diet [17]. A moderate-energy diet led to hyperphagia that was induced in dairy cows by the administration of FGF-21; this is indicative of FGF21-induced effects as a major regulator of feeding. Previous studies on changes in gene expression in the hypothalamus of FGF21-dosed DIO mice have shown that hyperphagia may occur via a compensatory nutrient, sensing-mediated process [18]. This response is due to FGF21-induced increases in energy expenditure due to the elevation in mRNAs for appetite-promoting hypothalamic neuropeptides, agouti-related peptide (AGRP) and nerve peptide Y (NPY). Indeed, these results indicate that FGF-21 exerts a direct influence on energy intake and also showe FGF-21 as an important endocrine and energy balance state regulator of metabolic homeostasis in dairy cows.

Here we showed that the relationship between serum FGF-21 levels and energy intake under conditions of negative energy balance. Our results demonstrate that serum FGF-21 levels increased significantly under NEB, induced by low-energy intake. Similarly, FGF-21 levels exhibited a long-term increase after parturition under NEB that was induced by a restriction of feed during late lactation. These results are consistent with our experimental findings, which showed that a lack of energy was the key factor that led to the increase in serum FGF-21 levels in early-lactation. This result also illustrates that FGF-21 levels are closely related to the regulation of energy metabolism. Our correlation analysis showed that FGF-21 levels had an inverse relationship with blood glucose. On the contrary, FGF-21 levels were not significantly related to insulin levels, which suggests that changes in FGF-21 levels is independent of variation in insulin levels. Previous experimental studies have similarly shown that FGF-21 can stimulate the uptake of non-insulin-dependent glucose [19] and that it has a positive impact on insulin sensitivity [20].

Current experimental research has shown that FGF-21 is a significant metabolic regulator that is produced primarily by the liver; it is necessary for normal activation of hepatic lipid oxidation, removal of triglycerides, ketogenesis induced by ketogenic diet, and adapting to fasting [21]. Our study demonstrated that serum FGF-21 levels and energy intake are closely related in dairy cows, and we also demonstrated the tendency of FGF-21 to vary under two different energy-state diets. The clarification of the molecular mechanism underlying the effects that FGF-21 plays in dietary interventions in NEB and its potential in the treatment of metabolic abnormalities in dairy cows with metabolic syndrome require further investigation.

## Conclusions

In summary, serum FGF-21 exerts a direct influence on energy intake levels, and serum FGF-21 significantly declines under high-energy diets and vice versa. We found that FGF-21 levels exhibited a significant positive correlation with serum leptin levels, while we found an inverse relationship between FGF-21 with blood glucose and BHBA levels.

## Abbreviations

NEB: Negative energy balance
FGF-21: Fibroblast growth factor-21
BHBA: β-hydroxybutyrate acid
DIO: Diet-induced obesity
TMR: Total mixed rations
Mcal: Mega-calorie
Glc: Glucose
TG: Triglyceride
NEFA: Non-esterified fatty acid
GC: Glucogon
INS: Insulin
LP: Leptin
GH: Growth hormone
BMI: Body mass index
NAFLD: Non-alcoholic fatty liver disease
AGRP: Agouti-related peptide
NPY: Nerve peptide Y
